# Comparative transcriptomic analysis of loquat floral fragrance and hormone synthesis regulation across developmental stages in petals and stamens

**DOI:** 10.3389/fpls.2025.1574771

**Published:** 2025-05-08

**Authors:** Jia-Qi Huang, Jia-Qi Wen, Fan Wu, Peng Zhou, Jing-Jing Zhang, Lin-Xuan Wang, Hong-Liang Li

**Affiliations:** Zhejiang Provincial Key Laboratory of Biometrology and Inspection & Quarantine, College of Life Sciences, China Jiliang University, Hangzhou, China

**Keywords:** *Eriobotrya japonica*, transcriptome, floral fragrance synthesis, hormone synthesis gene, real-time PCR

## Abstract

**Introduction:**

Loquat *Eriobotrya japonica* is a native plant in China that blooms at low temperatures in early winter, and the floral fragrance volatiles from the petals and stamens of loquats’ flowers are attractive to wild pollinators like Chinese honeybees. Thus, it was necessary to reveal the biosynthesis of floral fragrance and hormone regulation involved in the insect pollination of loquats’ flowers.

**Methods:**

Here, the volatile contents of petals and stamens were significantly higher than those of other parts of the loquat flower through the analysis of GC, and a key loquat flowers’ compound 4-methoxybenzaldehyde has the highest content among all volatile components. The transcriptomics of six samples of loquat flowers’ petals and stamens at different developmental stages of bud (Bu), exposed (Ex), and bloom (Bl) were obtained.

**Results:**

PCA analysis indicates that petals developed earlier than stamens due to the number of up-regulated petal genes being much higher than that of stamens in the bud stage, and the number of up-regulated stamen genes increasing rapidly at the stages of exposed and bloom. KEGG analysis revealed that petals and stamens DEGs were enriched in two pathways of plant hormone signal transduction and phenylpropanoid biosynthesis. Among them, some key genes related to the synthesis of the fragrance components were screened, and showing a strong positive correlation with phenethyl alcohol and 4-methoxybenzaldehyde. The synthesis of hormones such as gibberellin and growth hormone were also screened. Finally, real-time PCR was used to validate the screening of 12 genes related to floral fragrance and hormone synthesis. Except for *ACO* (1-Aminocyclopropane-1-carboxylate oxidase), most other genes located in the petals were expressed in significantly higher abundance than in the stamens. Among these, the expression of *PAAS* (Phenylacetaldehyde synthetase), *OMT* (O-methyltransferase), *GA2OX* (Gibberellin 2-*β*-dioxygenase) were consistent with the development of loquat flower.

**Discussion:**

Their high expression promoted the synthesis and release of floral fragrance and then may effectively attract pollinators. This study enriches the molecular mechanism of the release, synthesis and regulation of loquat floral fragrances and provides a theoretical basis for the co-evolutionary pollination between Chinese honey bees and loquat flowers in early winter.

## 1 Introduction

As a famous native fruit plant, the loquat *Eriobotrya japonica* is widely distributed around the Yangtze River basin in China. *E. japonica* commonly blossoms in late fall and early winter from October to February next year (Pinillos et al., 2011). Loquat mainly relies on insects of the bees (67.8%) and hoverflies (21.57%) for pollination, and these insects are essential for contributing fruits’ yield and quality (Saboor et al., 2021). During the period of blossom and pollination, the loquat flowers have relatively well-developed nectar glands, and emit distinctive and strong fragrances (Nyska et al., 2014). Nine loquat floral fragrance could be sensed by insect pollinators such as Chinese honey bees (Zhang, 2022), and the bees were more attracted to pollinate due to some specific fragrances at early winter temperatures (Huang et al., 2025). Although the main volatile components of loquat flowers have been characterized (Kuwahara et al., 2014), it was still unclear the mechanism of release, synthesis and regulation of the loquat floral fragrance during the winter blossom stage.

Floral fragrance was a significant means by which flowering plants attract pollinators and was intimately related to flowering behavior. Floral fragrance volatiles include terpenoids, phenylpropanoids, fatty acids, and amino acid derivatives (Natalia et al., 2013). The aromatic compound 4-methoxybenzaldehyde from loquat flower could efficiently bind with Chinese honey bees’ olfactory-related protein at low temperature (Zhang et al., 2023; Huang et al., 2025). It was reported to be synthesized by O-methyltransferase from loquat flowers (Koeduka et al., 2016a), and identified to be synthesized by benzaldehyde synthase in petunia flowers (Huang et al., 2022). The loquat floral volatile *β*-phenylethanol could also be attractive to Chinese honey bees at early winter (Zhang, 2022), and it was generated by decarboxylases located in the loquat flower (Koeduka et al., 2017).

On the other hand, the synthesis and release of plant floral fragrance could be affected by the regulation of plants’ flowering process (Huber et al., 2005; Su et al., 2022), some genes of which were involved in multiple signaling pathways for endogenous hormones and metabolism. For instance, the different bloom patterns regulate the growth hormone signaling genes (Guo et al., 2017), which could control the production of floral fragrances (Ke et al., 2019; Wang et al., 2021). Auxin-related proteins were involved in regulating flowering time (Zhu et al., 2020; Feng et al., 2022), and gibberellin-related genes were strongly associated with both floral longevity and bud unfolding (Yuan et al., 2024; Zhang et al., 2022b). Cytokinin-related genes regulate the development of female organs and inflorescence branching (Fu et al., 2022). However, these individual genes related to loquat’s floral fragrance synthesis and hormone regulations do not represent the whole process, which needs much more evidence to completely elucidate the synthesis and regulation pathway.

Comparative transcriptomic investigation has been extensively used in studies of floral development. For instance, the molecular mechanism of eggplant flowers’ anther dehiscence was identified using transcriptomics, and genes associated with another development were discovered (Yuan et al., 2021). The loquat has the ability of flowering in winter, it suggests that loquat has unique inner physiological mechanisms to adapt to low temperatures, and the blossom genes in whole loquat flowers have also been characterized by transcriptomics (Xia et al., 2020; An et al., 2021) and those key genes in the hormone signaling pathway (Jing et al., 2020). However, it was still unclear what the loquat flowers’ fragrance synthesis pathway and hormonal regulation were, which was crucial for the pollination biology of typical winter nectar plants like loquats.

Therefore, based on the GC characteristics of the fragrance of loquat flowers in developmental stages and different parts, this study investigates the transcriptomics of loquat flowers’ petals and stamens from various developmental stages, and analyzes the key genes that differ in expression, and the metabolic pathways to understand better the mechanism of fragrance synthesis and metabolism of loquat flowers. This study aims to reveal the pathways by which fragrance components and hormone synthesis were regulated during the development of loquat flowers. These findings help to interpret the molecular mechanism of loquat flower fragrance synthesis and provide new research viewpoints for the pollination biology of loquat flowers by flower-visiting insects.

## 2 Materials and methods

### 2.1 Plant materials

The materials used in this study were loquat flowers from the campus of China Jiliang University (30°19′17.25″N; 120°21′41.328″E). The flowers were collected from October to November 2023 and were categorized into three stages dependent on their morphology (Koeduka et al., 2016b): bud stage, exposed stage, and bloom stage.

### 2.2 Determination of loquat flowers’ volatiles by GC

**Figure 1:**
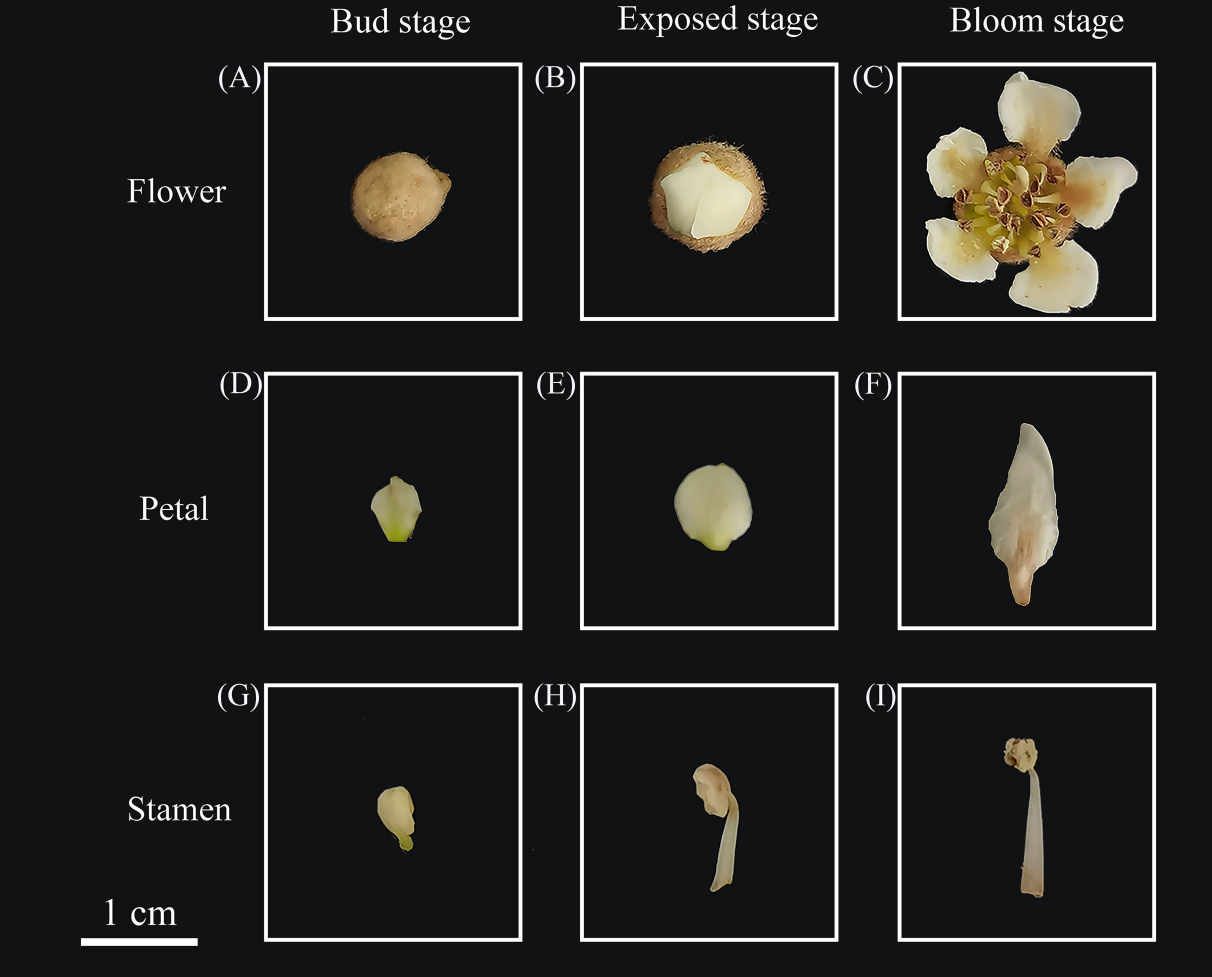
Loquat flowers, petals, and stamens from different stages. **(A)** loquat flower at the bud stage, **(B)** loquat flower at the exposed stage, **(C)** loquat flower at the bloom stage, **(D)** petal at the bud stage, **(E)** petal at the exposed stage, **(F)** petal at the bloom stage, **(G)** stamen at the bud stage, **(H)** stamen at the exposed stage, **(I)** stamen at the bloom stage.

Loquat flowers were collected at three stages (**Figures 1A–C**): bud stage (Bu), exposed stage (Ex) and bloom stage (Bl) as well as dissected petals and stamens, calyxes and pistils of loquat flowers at the bloom stage, and volatiles from loquat flowers at different stages were collected by headspace using a 65 μm PDMS/DVB SPME extraction column. The samples were placed in a 20 mL headspace vial and SPME adsorbed for 30 min at 30°C in a water bath. Standard samples of phenethyl alcohol, ethyl benzoate, 4-methoxybenzaldehyde, methyl 4-methoxybenzoate, ethyl 4-methoxybenzoate, (2-nitroethyl) benzene, methyl cinnamate, and (*E*)-ethyl cinnamate were diluted with methanol and analyzed by external standard using a gas chromatography GC-2014C (Shimadzu, Japan). The conditions of the GC analyses were as follows: using a Rtx^®^-1 (30 m×0.32 mm×0.25 μm, Shimadzu, Japan) column with nitrogen as the carrier gas at a flow rate of 1 mL/min. The inlet temperature was 250°C, the FID detector temperature was 280°C, and the column chamber warming program was set at 50°C for 2 min, and then the temperature was ramped up to 92.8°C at a rate of 20°C/min, and then ramped up to 95°C at 0.1°C/min, and finally ramped up to 150 °C at a rate of 20°C/min for a continuous period. The temperature was then increased to 150°C at a rate of 20°C/min for 10 minutes.

### 2.3 Total RNA extraction, library construction, and transcriptome sequencing

Fresh loquat flowers were collected from three different stages (**Figures 1A–C**), and the petals (**Figures 1D–F**) and stamens (**Figures 1G–I**) were carefully dissected with sterilized ophthalmic forceps. Furthermore, two biological replicates were set up for 12 samples, which were snap-frozen with liquid nitrogen and stored at -80°C in the refrigerator for subsequent transcriptome sequencing.

We commissioned Hangzhou Lianchuan Biotechnology Co., Ltd. to perform reference-free transcriptome sequencing of loquat flowers’ petals and stamens from different stages. Total RNA was extracted using the TRIzol method, and NanoDrop and Bioanalyzer evaluated its amount, purity and integrity. Qualified RNA (concentration >50 ng/μL, RIN >7.0, total amount >1 μg) was captured with oligo(dT) magnetic beads for polyA mRNA, fragmented, and reverse transcribed to cDNA. Double-strand synthesis was doped with dUTP, end-repairing plus A base and fragment ligations, and screening was performed. UDG enzyme treatment was followed by PCR amplification, resulting in a 300 bp ± 50 bp library. Finally, transcriptome data were obtained by double-ended PE150 sequencing using the Illumina Novaseq™ 6000 platform.

### 2.4 Transcriptome sequencing raw data processing

The downlinked data were in fastq format (Jing et al., 2022). The data were de-joined, de-low-quality, and repetitive sequences were removed to get the data formatted using fastq.gz, named Clean reads. The clean data reads were *de novo* assembled using Trinity to get the loquat flowers’ transcription group Unigenes database. Subsequently, the assembly quality of Unigenes was evaluated, including the length, Q20, Q30, and GC content of Unigenes.

### 2.5 Functional annotation of differential genes

Functional annotation of Unigenes was performed using the new comparison software DIAMOND, and six authoritative databases (NCBI_NR, GO, KEGG, Pfam, SwissProt, and eggNOG) were used for the annotation. The NCBI_NR, Pfam, SwissProt, and eggNOG databases retained all the best matches (those that satisfied the threshold value of 0.00001 and retained the smallest value). In contrast, the GO and KEGG databases retained all the annotations that satisfied the threshold value. Set the threshold of evalue 0.00001 and retain the annotation results with the smallest evalue, and GO and KEGG databases retain all the annotation results that satisfy the set threshold (evalue < 0.00001).

### 2.6 Functional enrichment analysis of differentially expressed genes

Using |log_2_FC|≥1 & FDR<0.05 as the criteria (no differential multiple for multi-group comparisons, and genes screened for FDR<0.05 as statistically significant differences among multi-groups). As a result, the genes screened for were considered differentially expressed genes (DEGs). GO (KEGG) functional significant enrichment analysis maps all significant differential expression Unigenes to each item, pathway of GO and KEGG annotation results of Unigenes. Additionally, using the hypergeometric test, the GO and KEGG annotation results of all the Unigenes were compared with the number of Unigenes for each entry and route. GO entries and KEGG pathways that were significantly enriched in differentially expressed Unigenes.

### 2.7 Expression trend analysis

In this study, STEM was utilized to distinguish different expression trends of genes during petal and stamen development in loquat flowers. The samples were set up according to the petal or stamen development stage, from bud to bloom. Selection of parameters: the data was processed using log normalization, and the STEM Clustering Method was employed for clustering (*p*<0.05). According to their expression patterns, the genes were divided into 16 modules, each representing a group of genes with comparable expression trends throughout the development of loquat flowers’ petals and stamens. The horizontal coordinates of the modules were Bu 1, Bu 2, Ex 1, Ex 2, Bl 1, and Bl 2, in that order, for a total of 6 samples. An inflection point represents a sample, and the vertical coordinate represents the change in gene expression. The genes in the expression module that showed an up-regulation trend were analyzed for KEGG pathway enrichment.

### 2.8 Validation of qRT-PCR for floral flavor and hormone-related genes

Six genes related to floral flavor and six genes related to endogenous hormones were screened and validated based on the enrichment results of the KEGG pathway, and the endogenous reference gene was *EjActin* (Jing et al., 2020). Primers were designed using Primer 5 (**Supplementary Table S1**). The RNA was reverse transcribed to obtain cDNA using a reverse transcription kit. Fluorescence quantitative detection was performed on a qRT-PCR instrument (Thermo Fisher Scientific, USA) using uGreener Fast qPCR 2×Mix reagent. The reaction program was as follows: pre-denaturation 95°C, 30 s; PCR reaction 95°C, 5 s; 55°C, 30 s; 40 cycles, and the samples were set up in three biological replicates using a 2^-^ *^δδCT^* to calculate the relative expression of differential genes.

### 2.9 Data statistics and analysis

Prism 8.0 was used to draw heat maps, and qPCR data were statistically analyzed. PCA, correlation analysis, Wayne plots, enrichment analysis, trend analysis, and clustering heatmaps of the transcriptome were done on the Lianchuan BioCloud platform (https://www.omicstudio.cn/tool).

## 3 Results

### 3.1 Analysis of volatiles in different stages and parts of loquat flowers

**Figure 2:**
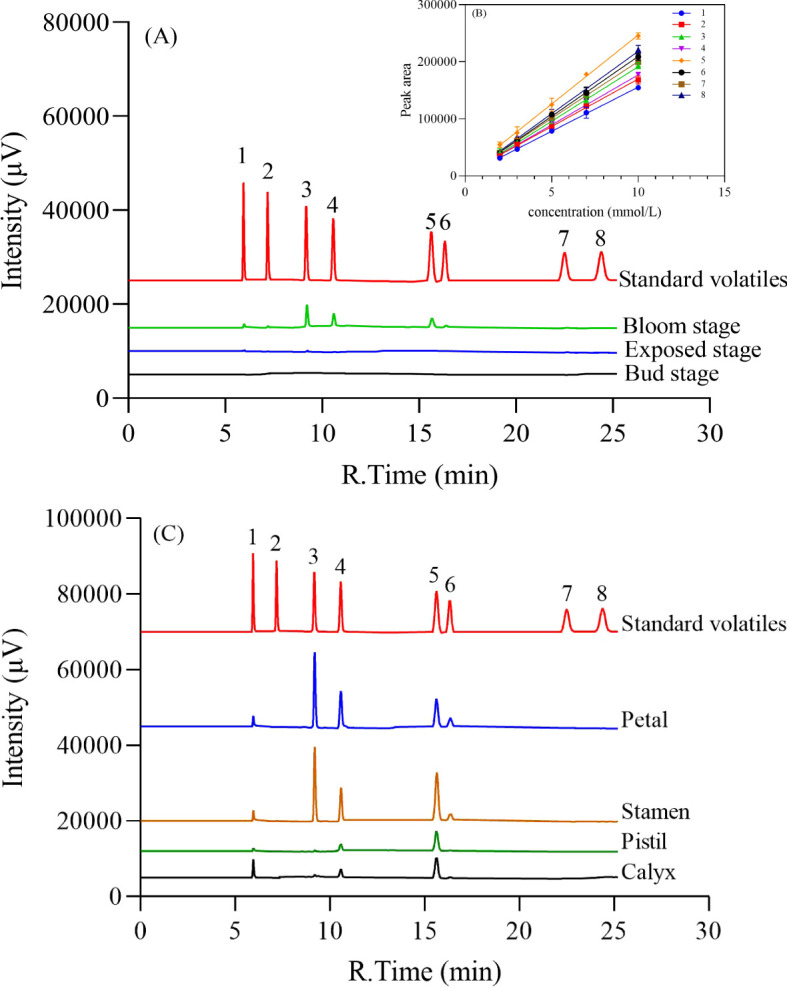
Analysis of the volatiles of loquat floral fragrance at different stages **(A)** and different parts of the bloom stage **(C)**. **(B)** Standard curve for standard volatiles. The standard volatiles are: (1) phenethyl alcohol; (2) ethyl benzoate; (3) 4-methoxybenzaldehyde; (4) (2-nitroethyl) benzene; (5) methyl 4-methoxybenzoate; (6) methyl cinnamate; (7) ethyl 4-methoxybenzoate; (8) (*E*)-ethyl cinnamate.

By the analysis of GC and external standard methods (**Figure 2B**), the volatiles of loquat flowers at different stages showed a trend of increasing during the developmental stages of loquat flowers (**Figure 2A**). Further analysis revealed that the volatile contents of petals and stamens were significantly higher than those of other parts of the loquat flower. It was found that 4-methoxybenzaldehyde has the highest content among all volatile components, which may be the key source of the unique fragrance of loquat flowers. In addition, all the three volatiles phenethyl alcohol, (2-nitroethyl) benzene and methyl 4-methoxybenzoate were detected in the loquat flowers’ four different parts (petals, stamens, pistils and calyxes). The distribution of methyl 4-methoxybenzoate (5) showed more popular than others among these four parts (**Figure 2C**).

### 3.2 Transcriptome sequencing and assembly quality assessment

Six samples from various loquat flowers’ parts at different stages of flowering—bud stage petals (Bu_p), bud stage stamens (Bu_s), exposed stage petals (Ex_p), exposed stage stamens (Ex_s), bloom stage petals (Bl_p), and bloom stage stamens (Bl_s)—were subjected to differential transcriptomics sequencing. The RNA-Seq data generated in this study (including samples from different developmental stages of petals and stamens) had been deposited in the SRA database, under accession numbers PRJNA1242521 and PRJNA1242523. The results showed 78.35G of raw data and 65.83G of valid data following preprocessing and assembly, with a valid data percentage above 83.25% (**Table 1**). The GC content ranged from 46.65% to 48.35%, and all Q20 and Q30 base proportions were over 98% and 95%, respectively. These results suggested that the transcriptome sequencing data matched the analytical standards and could be utilized for further research.

**Table 1:** The RNA sequencing quality of floral bud development in loquat.

Sample	Raw Data		Valid Data		Valid Ratio (Reads)	Q20%	Q30%	GC content%
	Read	Base	Read	Base				
Bu_p1	42163834	6.32G	36236400	5.27G	85.94	98.51	95.28	47.23
Bu_p2	42179608	6.33G	35429254	5.13G	84.00	98.54	95.33	47.62
Ex_p1	47905732	7.19G	39880960	5.79G	83.25	98.65	95.68	47.25
Ex_p2	45329948	6.80G	38073956	5.53G	83.99	98.64	95.63	47.40
Bl_p1	43832010	6.57G	36656518	5.31G	83.63	98.60	95.54	47.76
Bl_p2	45187518	6.78G	39032212	5.68G	86.38	98.44	95.06	47.94
Bu_s1	44076802	6.61G	39575002	5.76G	89.79	98.62	95.61	46.73
Bu_s2	40899348	6.13G	36538930	5.31G	89.34	98.64	95.67	46.65
Ex_s1	41061754	6.16G	37246576	5.44G	90.71	98.67	95.74	47.61
Ex_s2	41914152	6.29G	37722960	5.50G	90.00	98.68	95.76	47.80
Bl_s1	45742630	6.86G	39055454	5.67G	85.38	98.54	95.34	48.35
Bl_s2	42092074	6.31G	37387176	5.44G	88.82	98.68	95.76	47.83

### 3.3 Correlation analysis among transcriptome samples

PCA analysis separated the six sample groups into six clustering modules, as illustrated in **Figure 3**. Each sample group had distinct differences and specificity in gene expression levels. For example, the separation rates of PC1 and PC2 for different samples were 82.63% and 9.15%, respectively (**Figure 3A**). The PCA results showed that the data of the same group of samples were repetitive and differed between groups.

**BFigure 3:**
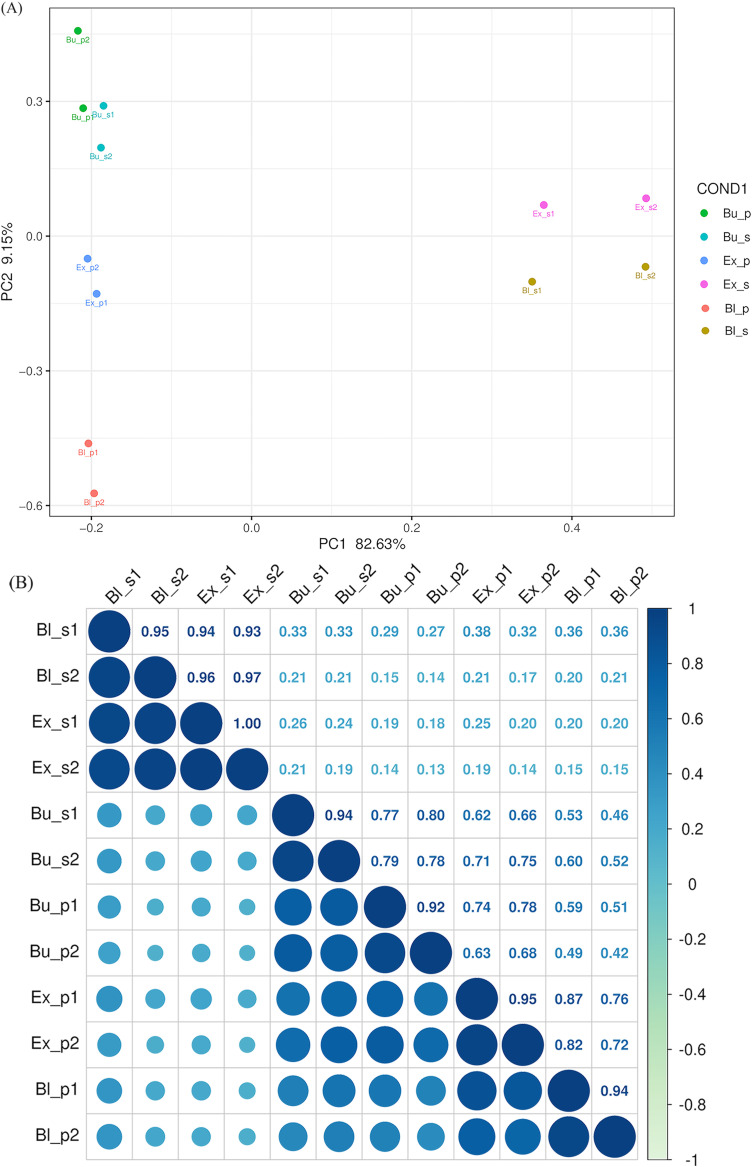
Principal component analysis and inter-sample correlation analysis of gene expression levels. **(A)** Principal component analysis showed similarity among replicate samples. **(B)** The inter-sample correlation coefficient, the clustering of 12 RNA-seq samples, represents the relationship between the two parts of the three stages. The depth of blue represents the similarity between the samples, and the darker the color, the higher the correlation. Bup, petal at the bud stage; Bus, stamen at the bud stage; Exp, petal at the exposed stage; Exs, stamen at the exposed stage; Blp, petal at the bloom stage; Bls, stamen at the bloom stage. The same as follows.

The inter-sample correlation coefficient analysis confirmed high sample similarity within each group, and the correlation coefficient tended to be approximately 1 (**Figure 3B**). Among them, the Ex_s and Bu_p groups demonstrated the most significant difference (Corr=0.16 ± 0.03). In contrast, the difference between the Bl_s and the Ex_s groups was negligible (Corr=0.95 ± 0.02).

The most incredible intergroup difference was between bud and bloom (Corr=0.50 ± 0.05), while the lowest was between bloom and exposed (Corr=0.79 ± 0.05) among loquat flowers’ petals at various stages. The difference between the exposed and bloom stages was the smallest (Corr=0.95 ± 0.01), while the contrast between bloom and exposed was the biggest (Corr=0.23 ± 0.02), comparing the stamens at different stages. Within the same stage, the bud stage correlated 0.78 ± 0.01, the exposed stage correlated 0.19 ± 0.03, and the bloom stage correlated 0.28 ± 0.07. Significant differences were discovered in the samples from different stages and parts of loquat flowers, indicating that the samples were well differentiated and taxonomically reliable.

A detailed functional annotation of the Unigenes was performed using the comparison tool DIAMOND. As indicated in **Table 2**, the annotation method encompassed six well-known database resources. Following analysis, 64645 and 60888 annotated Unigenes were discovered in the petals and stamens of loquat flowers, respectively. It offers a multitude of information for a thorough grasp of the molecular characterization of loquat blooms.

**Table 2:** Unigenes annotation information.

Part	Database of data	Number of Unigenes	Percentage/%
Petal	GO	36935	57.14
	KEGG	12726	19.69
	Pfam	30788	47.63
	swissprot	31802	49.19
	eggNOG	39312	60.81
	NR	38876	60.14
	All	64645	100.00
Stamen	GO	34405	56.51
	KEGG	12138	19.93
	Pfam	29448	48.36
	swissprot	29362	48.22
	eggNOG	36721	60.31
	NR	38235	62.80
	All	60888	100.00

### 3.4 Analysis of differentially expressed genes

**Figure 4:**
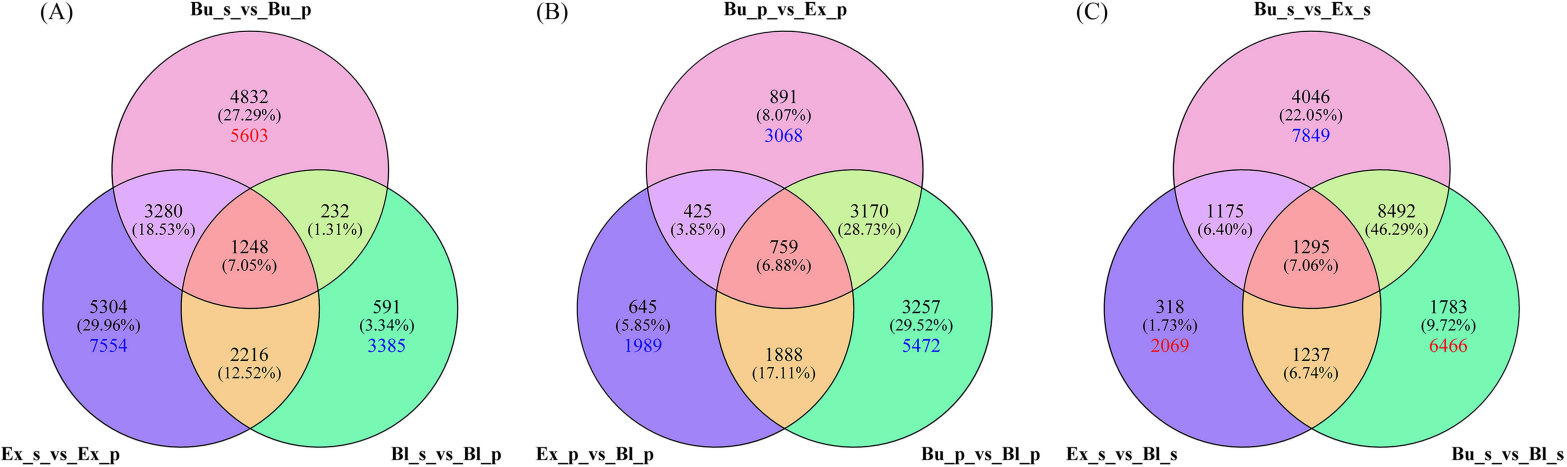
Venn diagram of genes expressed in different stages and locations of loquat flowers. **(A)** Venn diagram of differentially expressed genes between stamens and petals in the same stage. **(B)** Venn diagram of differentially expressed genes in petals at different stages. **(C)** Venn diagram of differentially expressed genes in stamens at different stages. The crossing points indicate the number of common genes. Red and blue numbers indicate the number of up-regulated and down-regulated DEGs in the corresponding pair.

With high-quality transcriptome data and screening criteria, we examined up- and down-regulated DEGs in petals and stamens from the same stage and petals or stamens from different stages. In petals and stamens of the same stage, 9592, 12048, and 4287 DEGs were detected between Bu_s and Bu_p, Ex_s and Ex_p, and Bl_s and Bl_p, respectively. Among them, 5603 DEGs were up-regulated and expressed between Bu_s and Bu_p, 7554 DEGs were down-regulated and expressed between Ex_s and Ex_p, and 3385 DEGs were down-regulated and expressed between Bl_s and Bl_p (**Figure 4A**).

In petals at different stages (**Figure 4B**), 5245 (with 3068 genes down-regulated in the expression), 3717 (with 1989 genes down-regulated in the expression), and 9074 (including 5472 genes down-regulated in the expression) were detected in the comparisons between Bu_p vs. Ex_p, between Ex_p vs. Bl_p, and between Bu_p vs. Bl_p, respectively. Among the stamens in different stages, Bu_s vs. Ex_s had the down-regulated DEGs with 7849, 2069 up-regulated DEGs between Ex_s vs. Bl_s, and 6466 up-regulated DEGs between Bu_s vs. Bl_s (**Figure 4C**).

### 3.5 Functional enrichment of differentially expressed genes

**Figure 5:**
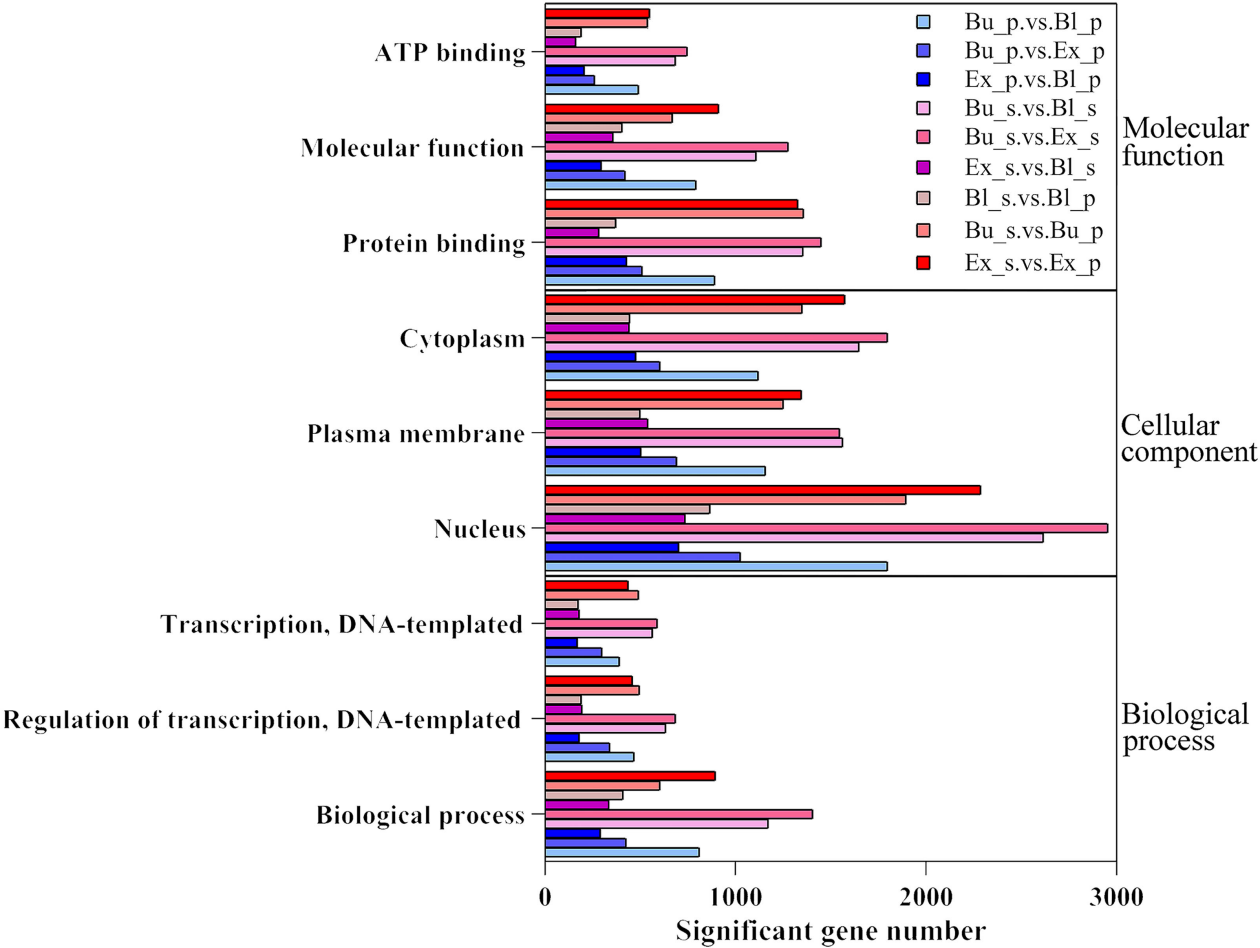
GO enrichment analysis of differentially expressed gene.

GO enrichment analysis was performed for different groups, and results were displayed in **Figure 5**. The results were grouped based on molecular function (MF), biological process (BP), and cellular components (CC). The enrichment analysis histogram showed that most differentially expressed genes were enriched in the biological process (BP) entries. The primary enrichment was in the biological process, regulation of transcription, DNA-templated and transcription, DNA-templated. Cellular component (CC) and molecular function (MF) come next; the former was primarily enriched in the cytoplasm, plasma membrane, and nucleus, while the latter was enriched mainly in ATP binding, protein binding, and molecular function. Among them, the Bu_s vs. Ex_s group DEGs had the comparatively most number among the entries.

**Figure 6:**
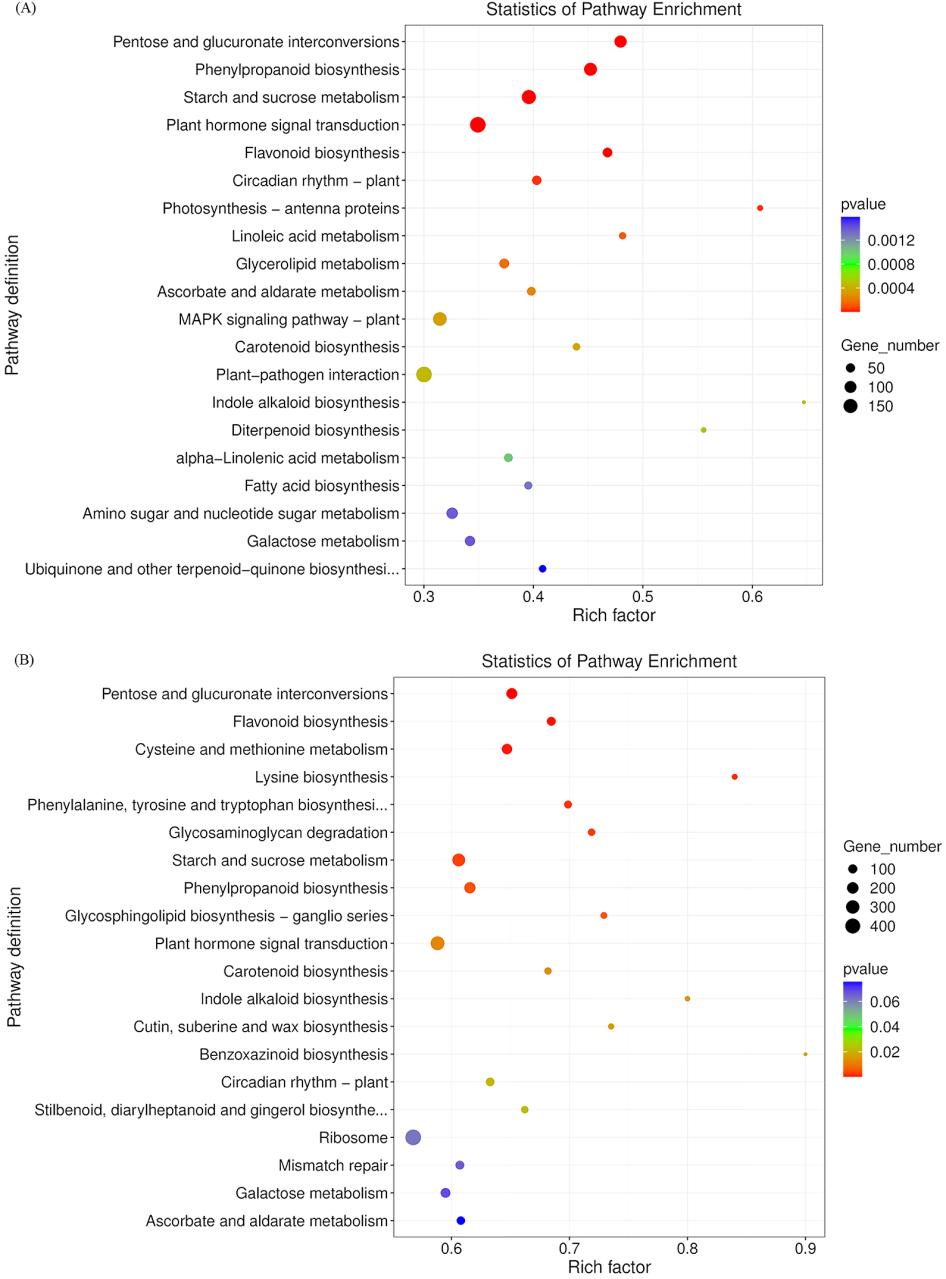
KEGG enrichment analysis of differentially expressed genes in petals **(A)** and stamens **(B)** at different stages.

To visualize DEGs in metabolic pathways, we classified these DEGs according to KEGG pathway enrichment analysis. The Bu_p vs. Ex_p vs. Bl_p DEGs were mainly enriched in plant hormone signal transduction (197), starch and sucrose metabolism (154), phenylpropanoid biosynthesis (123), pentose and glucuronate interconversions (106) and glycerolipid metabolism (59) (**Figure 6A**). The DEGs of Bu_s vs. Ex_s vs. Bl_s were mainly enriched in plant hormone signal transduction (320), starch and sucrose metabolism (254), phenylpropanoid biosynthesis (181), pentose and glucuronate interconversions (168), and cysteine and methionine metabolism (143) (**Figure 6B**). In conclusion, DEGs from petals and stamens of loquat flowers at different stages were enriched in pathways of plant hormone signal transduction, starch and sucrose metabolism, and phenylpropanoid biosynthesis.

### 3.6 Trend analysis of differentially expressed genes

**Figure 7:**
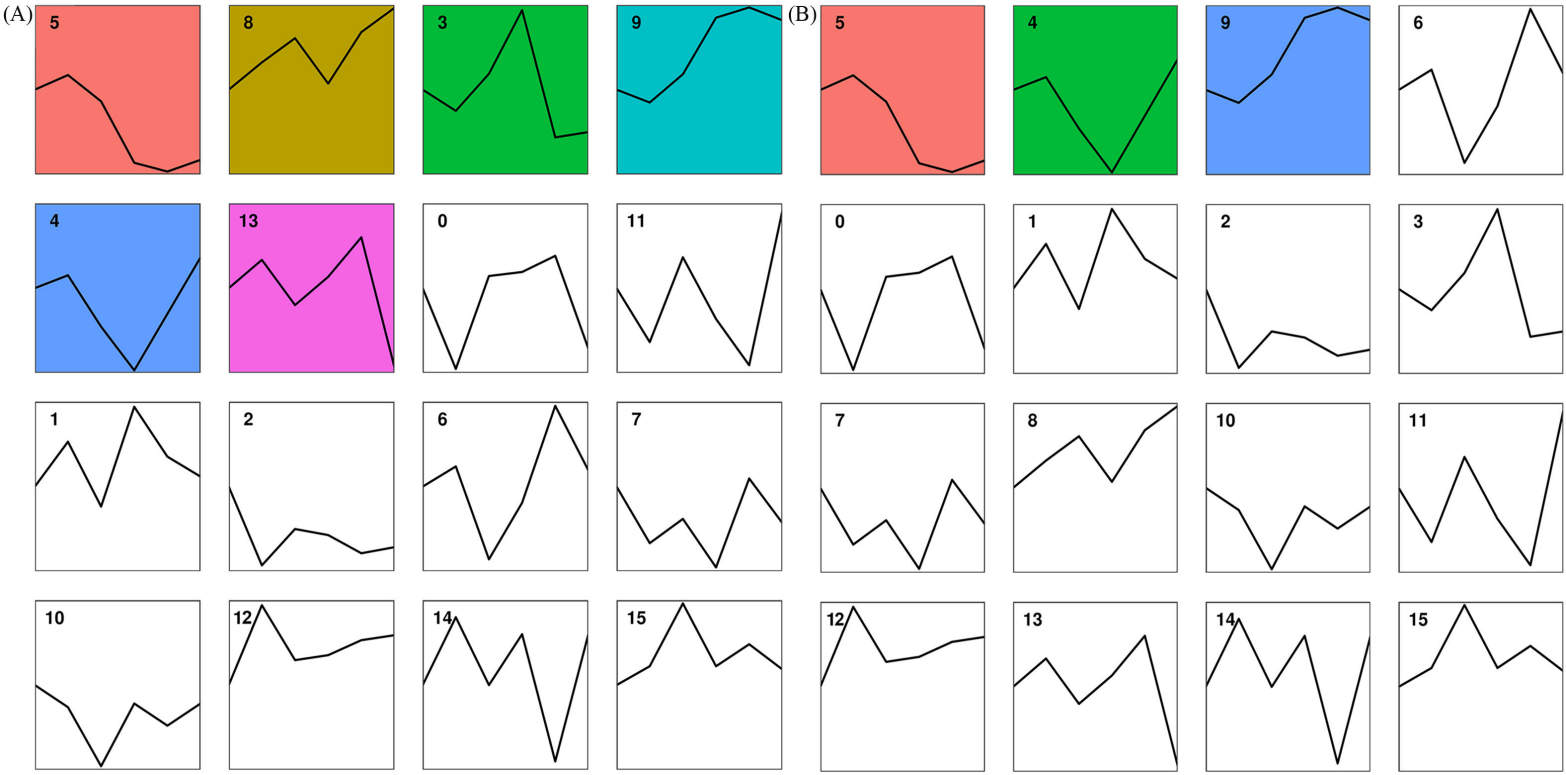
Plot of gene expression trends at different developmental stages in petals **(A)** and stamens **(B)**. The line graph trend represents the overall trend of gene expression in this cluster over time. Significant clusters are highlighted with a colored background. The number at the top left of each line graph was the name of the cluster.

**Figure 8:**
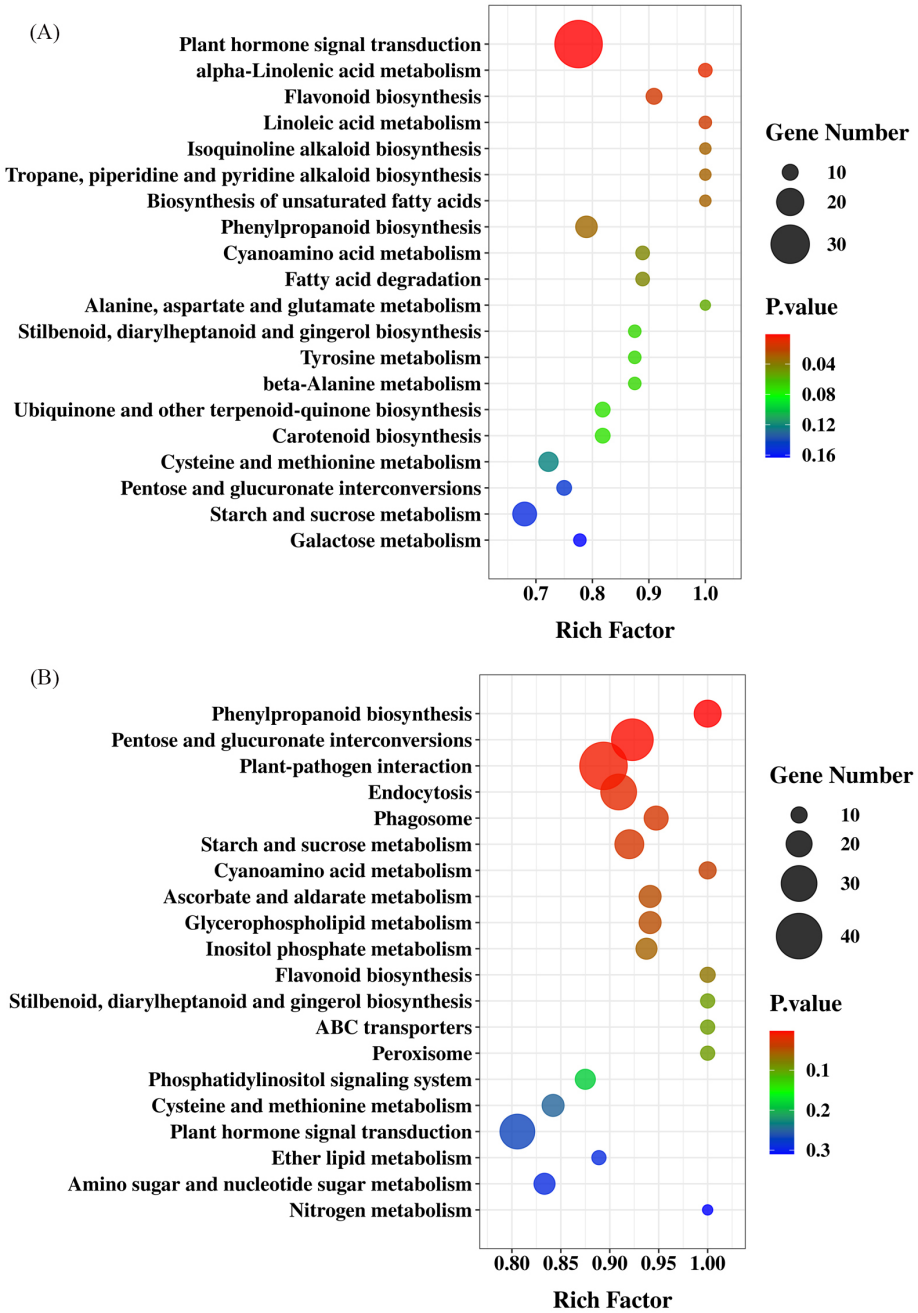
Metabolic pathways significantly enriched in petal **(A)** and stamen **(B)** in profile 9.

DEGs from petals were assigned to 16 trends (**Figure 7A**). Among them, Profile 5, Profile 8, Profile 3, Profile 9, Profile 4, and Profile 13 were significant trends. While the genes in Profile 9 indicated a continuous up-regulation trend from the bud stage to the exposed stage to the bloom stage, the genes in Profile 5 indicated a continuous down-regulation trend with the development of loquat flowers. After comparing the genes in Profile 9 with the KEGG database, 721 of the 3266 genes were annotated. Illustrated in **Figure 8A**, they were primarily considerably enriched in flavonoid biosynthesis (10), phenylpropane biosynthesis (15), and phytohormone signaling (38).

DEGs from stamens were also assigned to 16 trends (**Figure 7B**). Profile 5, Profile 8, Profile 3, Profile 9, Profile 4, and Profile 13 were significant trends. The trends for Profile 5 and Profile 9 were the same as in Petals. The genes in Profile 9 were compared with the KEGG database; 738 of the 3068 genes were annotated in the KEGG database. As shown in **Figure 8B**, the primary highly significant enrichment was in plant-pathogen interactions (42), pentose and glucuronate interconversions (36), and phenylpropanoid biosynthesis (21).

### 3.7 Synthetic related genes of floral fragrance and hormone

**Figure 9:**
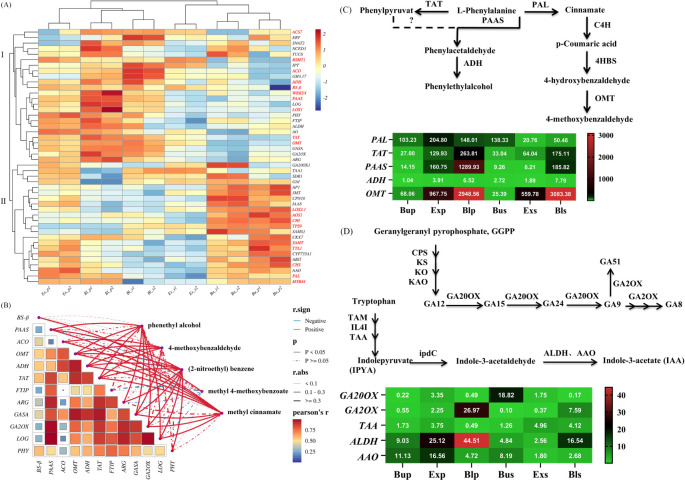
Gene heatmap of petals and stamens of loquat at different stages. **(A)** Hierarchical clustering analysis of key candidate DEGs for floral fragrance and hormones. Z-score standardized gene expression level data. Red and blue represent up-regulated and down-regulated genes, respectively. Genes related to floral fragrance synthesis (red annotation); genes related to hormones (black annotation). **(B)** Correlations between genes and volatile compounds. The red and blue solid lines separately indicate positive and negative correlation, and the dotted line indicates a non-significant correlation (*p*≥0.05). The higher the correlation coefficient (r. abs, absolute value), the thicker the line. The correlation coefficient of the heatmap is Person’s r, red represents the higher *r* values. Gene expression patterns associated with floral fragrance volatiles **(C)** and hormone anabolism **(D)**. The corresponding synthetic pathways of phenethyl alcohol and 4-methoxybenzaldehyde, gibberellin (GA) and auxin (IAA) were analyzed at different developmental stages of petals and stamens. Successive arrows indicate one-step enzymatic reactions, and dashed lines indicate unknown enzymes. In the heat map, red and green indicate maximum and minimum values, respectively; each line was independent.

Functional annotation, functional classification, and metabolic pathway and trend analysis of loquat flowers’ transcriptome sequencing results revealed that 19 and 27 DEGs related to floral fragrance and endogenous hormones were screened, respectively. Heat map clustering analysis could reflect the different gene expressions of loquat flowers’ petals and stamens in different stages. As shown in **Figure 9A**, the gene expression of loquat flowers’ petals and stamens was divided into 2 clusters. Cluster I had a higher expression in Ex_p, Bl_p, and Bl_s, and Cluster II had a higher expression in Bu_p and Bu_s. Cluster I was only sparingly expressed in the bud stage of early development, but it increases dramatically following the development of petals and stamens into the exposed and bloom stages. In contrast, the expression of cluster II peaked at the early stage of flower development. It was maintained relatively low as the flowers developed during the exposed stage.

The correlation network diagram was drawn to illustrate the significant correlations between different genes and specific volatile compounds. As **Figure 9B** shown, *OMT* (O-methyltransferase), *ADH* (Alcohol dehydrogenase), *TAT* (Tyrosine aminotransferase), and *GASA* (Gibberellic acid-stimulated in Arabidopsis) showed a strong positive correlation (labelled by solid red line) with phenethyl alcohol, 4-methoxybenzaldehyde, (2-nitroethyl) benzene, and methyl cinnamate. The *r* values of the *OMT* with 4-methoxybenzaldehyde and *PAAS* (Phenylacetaldehyde synthase) with phenethyl alcohol were 0.914 and 0.616, respectively. Notably, methyl 4-methoxybenzoate was only positively correlated with *BS-β*, with an *r* value of 0.769 (**Figure 9B**).

In this study, *PAAS* and *OMT* were discovered among 19 up-regulated DEGs associated with synthesizing loquat floral fragrance volatiles. Here, *PAAS*, being the highest expression in petals during the bloom stage, was associated with phenylacetaldehyde synthesis. *OMT*, associated with synthesis of 4-methoxybenzaldehyde, was highly expressed in the stamens and petals during the bloom stage (**Figure 9C**). Similarly, based on the screening among the 27 up-regulated DEGs linked to loquat hormone anabolism, *GA2OX* (Gibberellin 2-*β*-dioxygenase) about the synthesis of gibberellin, showed strongly expressed in bloom petals. The growth hormone-related *ALDH* (Aldehyde dehydrogenase) was substantially expressed in both bloom petals and stamens (**Figure 9D**).

### 3.8 Validation of differential expression gene by qRT-PCR

**Figure 10:**
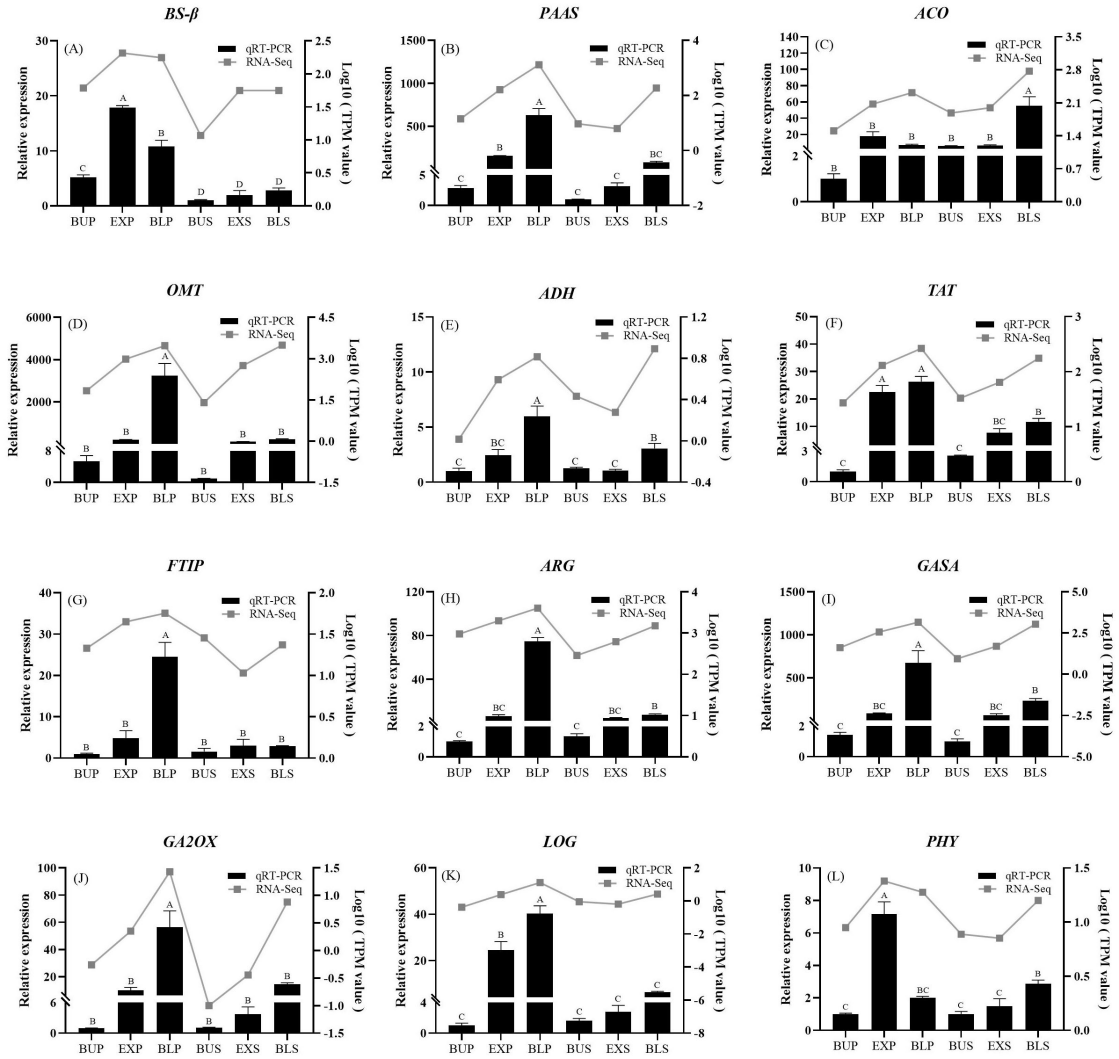
qRT-PCR analysis of differentially expressed genes. (*p*<0.01).

Six floral and six hormone anabolism-related DEGs were screened from the transcriptome results. The qRT-PCR was used to validate the expression changes of these 12 genes (**Figure 10**). The results demonstrated that the relative expression patterns of these genes aligned with the transcriptome sequencing results. The results of the transcriptome data were confirmed to be credible. All the genes except *EjACO* (1-Aminocyclopropane-1-carboxylate oxidase) had higher expression in petals than in stamens. The floral fragrance-related genes, *EjPAAS*, *EjOMT*, *EjTAT*, and hormone-related genes, *EjFTIP* (FT-Interacting protein), *EjARG* (Auxin-induced protein), *EjGASA*, *EjGA2OX*, and *EjLOG* (Lonely·guy), were all increased in expression with the development of petals and stamens. It indicated that they might have a tight connection to the development process of petals and stamens.

## 4 Discussion

### 4.1 The floral fragrance of petals and stamens at the bloom stage

Flowering plants release different fragrance components at different flowering stages and the fragrance of flowers generally gradually increases. In this study, the loquat floral fragrance showed the highest release at the bloom stage (**Figure 2A**). This highest release of fragrance was able to attract pollinators for effective visiting and nectar collecting (Koeduka, 2018). It was found in the *Paeonia lactiflora* (Zhao et al., 2024) that the release of monoterpenoids was highest at the S3 stage, i.e., the full blooming stage, which was consistent with the increase in the intensity of floral fragrance in the senses, suggesting that the peak stage of floral fragrance corresponded to the blooming stage of flowers.

Most studies reported that floral fragrance in plants were mainly released from petals, but other floral organs such as stamens, pistils, calyxes and nectar glands also contribute partially to fragrance (Gerard et al., 2013). In this study, it was found that the fragrance components of loquat flowers primarily came from the petals and stamens (**Figure 2B**). Similarly, other studies have found that the volatile components accumulated in the stamens of *Camellia* were much higher than those of petals (Jullien et al., 2008). In terms of five identified loquat floral volatile components in this study, the previous similar reports showed that the major loquat flowers’ fragrances were (2-nitroethyl) benzene, 4-methoxybenzaldehyde and methyl 4-methoxybenzoate, while phenethyl alcohol were the minor components (Kuwahara et al., 2014; Watanabe et al., 2021; Koeduka et al., 2016a).

### 4.2 DEGs of petals and stamens in different developmental stages

The plant flowering process was significantly regulated by the related hormones and flowering genes, which has been found in the development of loquat terminal buds, several genes controlling flowering timeof which were related to the ABA signaling pathway (An et al., 2021). Some candidate genes might regulate loquat flowering by exogenous gibberellin treatment and proposed a hypothetical model for loquat flowering regulation (Jiang et al., 2021). Furthermore, transcriptome analysis of loquat from vegetative apex to flower bud transition revealed that the *EjAGL17* gene was flowering gene that significantly up-regulated at the flower bud transition stage to promote loquat in blossoming (Xia et al., 2020). Nevertheless, the genes related to synthesis and release of plant floral voltiles were also involved in the present transcriptome analysis.

In this study, we obtained the DEGs from petals and stamens from three different stages of loquat flowers by the analyis of transcriptome sequencing. It revealed that the number of up-regulated genes in petals was much higher than that in stamens during the bud stage (**Figure 4A**). Moreover, the number of up-regulated genes in stamens increased rapidly as the flowers developed into the exposed and blooming stages, suggested that petals develop earlier than stamens. Therefore, it was hypothesized that loquat flowers’ petals might be the first to release floral fragrance, enabling insects to detect the loquat flowers earlier. It might extend the time for insects to visit the flowers and enhance the pollination efficiency of loquat flowers.

In addition, the number of genes in the petals was significantly higher between the bud and bloom stages than in the other two groups (**Figure 4B**). It may be involved in petal expansion, pigment accumulation, and the synthesis of fragrance compounds. There were more down-regulated genes than up-regulated genes in petals at the bloom stage, probably because petals at the bloom stage no longer require genes with higher expression levels. On the contrary, the down-regulation of genes may promote petal apoptosis, and petals begin to enter the senescence phase. It has been reported that the total number of up-regulated and down-regulated genes was the highest in both the bud stage (FBE) and the bloom stage (FA) of loquat flower development (Jing et al., 2020). It was consistent with the results of this research, which showed that the highest intensity of gene expression regulation was throughout the bud and bloom stages of loquat flower development.

Interestingly, this study discovered a more significant number of total up-and down-regulated genes between the bud and exposed stages of stamens (**Figure 4C**). This implies that the dynamics of gene expression were more dramatic during these two stages, which could also be connected to the bud stage’s development of stamens and pollen grain formation (Maura and Valentina, 2014).

### 4.3 Key pathway enrichments in hormone and floral fragrance synthesis

This study conducted a GO enrichment analysis of loquat flowers at different developmental stages (**Figure 5**). The results indicate that loquat flowers play a key role in cellular energy metabolism, signal transduction, and protein stability. Other studies have also pointed out that loquat gene expression was concentrated in cellular and metabolic processes, and was more abundant in membranes, cells and organelles, mainly involving binding, catalytic activity, and transport protein activity (Zhang et al., 2022a), which was consistent with the findings of this study.

The results showed that the DEGs in loquat petals and stamens were mainly enriched in the KEGG pathway in plant hormone signal transduction, starch and sucrose metabolism, phenylpropanoid biosynthesis (**Figure 6**), and other related research results also verified this point (Jing et al., 2020; Zhang et al., 2022a), indicating that these metabolic pathways were important for loquat flower development.

In this study, we further clarified the role of different parts in the development of loquat flowers, and found that the expression of up-regulated gene in petals and stamens (**Figure 7**), which may be involved in the synthesis of aromatic compounds in petals and pollen maturation. Plant hormone signaling was significantly enriched in the gene expression trend of petals (Jing et al., 2020), and it was speculated that it may regulate the production of floral flavor volatiles. The biosynthesis of phenylpropanoid (Ramya et al., 2017) and flavonoids affects the color of petals and stamens and their attraction to pollinating insects, while changes in glucose metabolism in stamens were associated with energy regulation.

### 4.4 Key genes in floral fragrance synthesis

The floral fragrance was a natural mechanism by which plants attract pollinators. Floral fragrance consists of low molecular weight volatile organic compounds (VOCs), typically produced by plants’ secondary metabolic pathways (Natalia et al., 2013). This study discovered many phenylpropanoid biosynthesis-related genes in loquat flowers’ petals and stamens (**Figure 8**) in the top three pathways. The specific loquat floral fragrance composition (2-nitroethyl) benzen was reported to be oxidized by CYP94A90, and produced from *L*-phenylalanine as a precursor (Yamaguchi et al., 2021; Kuwahara and Asano, 2018). Based on the GC (**Figure 2**) and transcriptome (**Figure 9**), *PAAS* (*AADC*), *OMT*, and *ADH*, having the highest expression in petals at the bloom stage verified by qPCR experiments (**Figure 10B**, 10D, 10E), were positively correlated with volatile release (**Figure 9B**).

Among them, the *EjAADC1* was a key gene controlling the biosynthesis of volatile benzene compounds in flowers (Koeduka et al., 2017). *EjAADC1* can convert *L*-phenylalanine into phenylacetaldehyde, and was expressed explicitly in petals (Koeduka et al., 2017). Similarly, the expression of *PAAS* in *Murraya paniculata* was higher than that in *Citrus maxima*, with the function of phenylacetaldehyde synthesis (Yang et al., 2023). For the expression pattern of *EjOMT*, it was consistent with the changes of volatile benzoates in the floral organs (**Figure 10D**). That indicates that *EjOMT1* has broad substrate specificity for compounds with *p*-hydroxy and *o*-methoxy groups (Koeduka et al., 2016a). In addition, the ADH family was involved in the interconversion of various alcohols and aldehydes, including phenylacetaldehyde (Strommer, 2011). Therefore, these key genes identified in this study, *PAAS*, *OMT*, and *ADH*, were expressed in the petals of loquat flowers at the complete bloom stage higher than in the stamens. It suggests that they make a great contribution of efficiently synthesizing volatile fragrance compounds in the petals and thus attracts pollinators like bees.

In this study, we discovered that *EjBS-β* expression of benzaldehyde synthase was significantly higher in the petal exposed stage than in the bloom stage (**Figure 10A**). Benzaldehyde synthase was a heterodimeric enzyme consisting of two subunits, *α* and *β*, which catalyze the synthesis of benzaldehyde in the presence of both subunits together (Huang et al., 2022). The significant up-regulation of the benzaldehyde synthase gene may resulte in the synthesis and accumulation of benzaldehyde in the petal exposed stage, and provide the base for the other derivatives in the following blooming stage (Wickramasinghe and Munafo, 2020). Therefore, the high concentrations of benzaldehyde and the derivatives were released during the flowering period to attract pollinators such as bees.

The ethylene biosynthesis gene *EjACO* was also discovered to be expressed at a significantly higher level in loquat flowers’ stamens than in petals (**Figure 10C**). It implies that the cells of the stamen part may have a higher demand or utilization efficiency for ethylene synthesis during loquat flowers’ stamen development. The same example was discovered in carnations that flower senescence of carnations was regulated by endogenous ethylene (Norikoshi et al., 2022). In another study, one of four *ACO* genes in tomato flowers had the highest expression and prompted the development of the petals and pistils (Llop-Tous et al., 2000).

The expression of tyrosine aminotransferase *EjTAT* was significantly higher in petals than in stamens during the dewlap and bloom stages (**Figure 10F**). It has been reported in loquat, which may produce methyl 4-methoxybenzoate, 4-methoxybenzaldehyde, etc., through the metabolic pathway of tyrosine (Kuwahara and Asano, 2018). The exposed stage may be when loquat blossoms get ready to bloom to attract pollinators.

### 4.5 Key genes in hormone-related

This study discovered that the number of differential genes engaged in plant hormone signal transduction was first in both petals and stamens (**Figure 6**). The synergistic action of endogenous hormones and flowering genes was critical to regulating blossoming in loquat (Chi et al., 2020). The emission of floral fragrances was indirectly influenced by phytohormones, which were crucial in controlling flower development (Chandler, 2011).

In this study, we also discovered a positive correlation between hormone genes and volatiles (**Figure 9**), and the expression of hormone genes increases with the maturation of loquat flowers. For example, the flowering hormone-associated effector protein *EjFTIP* and *EjGA2OX* were increased only in petals, and its expression in stamens was insignificant (**Figures 10G, J**). In addition, *OsFTIP1* has also been reported to be primarily involved in regulating the flowering time in rice. It was discovered that the degree of late flowering in *OsFTIP1* RNAi plants was mainly correlated with the down-regulated level of *OsFTIP1* expression (Song et al., 2017). And it was reported that *JcGA2OX6* had a great impact on the vegetative and reproductive growth of plants (Hu et al., 2017). Therefore, our results suggested that *EjFTIP* and *EjGA2OX* may have a more pronounced impact on petal development compared to stamens.

*EjARG* and *EjGASA* were significantly increased in petals and up-regulated in stamens, with the lowest expression at the bud stage (**Figures 10H, I**). Similar studies in roses showed that the expression of *ARG* was up-regulated during flowering to promote flowering (Guo et al., 2017). And in *Prunus mume* (Zhang et al., 2022b), nine *PmGASA* have been discovered to be significantly increased in expression during the bud opening stage. As regulators of loquat blooming time, bud differentiation, and flower development, *PmGASA* might be crucial to the flowering process of flower buds. Thus, *EjARG* and *EjGASA* were commonly associated with the flowering transition in loquat.

Only three petal stages in the current study showed considerable up-regulation of *EjLOG* (**Figure 10K**), suggesting that *EjLOG* was crucial for petal development. Key genes *LOG1*, *LOG3*, and *LOG7* in the cytokinin and gibberellin pathways of chestnuts (Wu et al., 2022) have been reported to affect female flower formation. In this study, *EjPHY* (Phytochrome) was discovered to have the highest expression in petals at the exposed stage (**Figure 10L**). The *PHYB* and *PHYC* were also discovered to promote flowering through the photoperiodic pathway in *Triticum aestivum* (Stephen et al., 2016). According to the information above, photosensitive pigments control when flowers bloom during the exposure stage. Petals may need to regulate PHY expression to manage flowering timing and maximize pollination prospects.

## 5 Conclusion

In this study, GC analysis showed that the fragrance of loquat flowers was primarily released in the blossom stage and from its petals and stamens. The comparative transcriptomic analysis of petals and stamens in the different developmental stages showed that the development of petals occurs earlier than that of stamens, and the changes in gene expression during the early and mid-stages of stamens were more drastic. The KEGG enrichment analysis revealed that the DEGs were primarily enriched in the plant hormone signal transduction pathways related to endogenous hormones and the phenylpropanoid biosynthesis pathways associated with floral fragrance synthesis. *PAAS* and *OMT* related to synthesis of key loquat floral volatiles were screened from 19 up-regulated DEGs associated with synthesizing loquat floral fragrance volatiles. *GA2OX* and *ALDH* related to gibberellin and growth hormone were screened from 27 up-regulated DEGs linked to loquat hormone anabolism, respectively. The expression of floral fragrance and hormone synthesis genes in petals and stamens at different developmental stages is positively correlated with the content of volatiles. Finally, the expression of 11 candidate genes (5 and 6 genes related to floral volatiles and hormone synthesis, respectively) were validated by qRT-PCR.

## Data Availability

The datasets presented in this study can be found in online repositories. The names of the repository/repositories and accession number(s) can be found in the article/**Supplementary Material**.
