# Supplementary material for: Comparative transcriptomic analysis of loquat floral fragrance and hormone synthesis regulation across developmental stages in petals and stamens
Source: Front Plant Sci. 2025 May 8;16:1574771. doi: 10.3389/fpls.2025.1574771 (PMC12095192; doi:10.3389/fpls.2025.1574771)
Supplement: Supplementary file 1 [file Table1.docx]

Table S1 Primer sequences used for qRT-PCR

| Gene classification | Gene | Gene name | Genbank | Primer (5'-3') |
| --- | --- | --- | --- | --- |
| Internal reference gene | *Ejactin* | *β*-actin | XM_028353190.1 | AATGGAACTGGAATGGTCAAGGC |
|  |  |  |  | TGCCAGATCTTCTCCATGTCATCCCA |
| Floral fragrance related genes | *EjBS-β* | Benzaldehyde synthase-*β* | XM_050279684.1 | GAATAGAGAGAGCGAAGAAGC |
|  |  |  |  | TATATGACAGTTGAAGCCAGA |
|  | *EjPAAS* | Phenylacetaldehyde synthase | LC310891.1 | GCAAATAGCCTTGAGCCGAAG |
|  |  |  |  | GGCGAAACCCTAAAACAGACC |
|  | *EjACO* | 1-Aminocyclopropane-1-carboxylate oxidase | GQ377219.3 | GCAACTACCCTCCATGTCCC |
|  |  |  |  | CATCAACCCACTCTCCGTCC |
|  | *EjOMT* | O-methyltransferase | LC127201.1 | TTCAACTDGGCATCCCAGA |
|  |  |  |  | CTCCACATCGTCACAATCA |
|  | *EjADH* | Alcohol dehydrogenase | XM_068486657.1 | ATCTCTTCCCTGCTCTCAAT |
|  |  |  |  | ATACACAACACCCAAAGTCTC |
|  | *EjTAT* | Tyrosine aminotransferase | XM_029089035.2 | GCAGACGGCTGATTTCTTTGGG |
|  |  |  |  | TTGAACTTGTCGGACTGGAGGG |
| Hormone related genes | *EjFTIP* | FT-Interacting protein | XM_009364164.3 | TTGATCCTTATGTGGAGGT |
|  |  |  |  | CTTGTCTTTGACGGTGACT |
|  | *EjARG* | Auxin-induced protein | XM_050256654.1 | GTGGATATGCGGCGGGTTC |
|  |  |  |  | CGTTTACGCTTCTGGGAGC |
|  | *EjGASA* | Gibberellic acid-stimulated in Arabidopsis | XM_008389727.4 | CTCGTTTCCCTTCTTGTTTTC |
|  |  |  |  | CCCCACCACAGTCTATTTTCTT |
|  | *EjGA2OX* | Gibberellin 2-*β*-dioxygenase | XM_068466155.1 | ACAACACCGTCAACAAAGCC |
|  |  |  |  | CACTACCTGAAAGAAACCCCACT |
|  | *EjLOG* | Lonely·guy | XM_068482606.1 | GGAAGTAGTCCAGGAAACAAAAG |
|  |  |  |  | GTAAGGCAATAAAGGCATCAGAG |
|  | *EjPHY* | Phytochrome | XM_050287059.1 | GCCCCACACAGTTGCCATTTAG |
|  |  |  |  | AACCTCGTCATCCGCCTCAT |
